# Amyand's hernia-a vermiform appendix presenting in an inguinal hernia: a case series

**DOI:** 10.1186/1752-1947-5-463

**Published:** 2011-09-19

**Authors:** Kyriakos Psarras, Miltiadis Lalountas, Minas Baltatzis, Efstathios Pavlidis, Anastasios Tsitlakidis, Nikolaos Symeonidis, Konstantinos Ballas, Theodoros Pavlidis, Athanassios Sakantamis

**Affiliations:** 1 2nd Propedeutical Department of Surgery, Hippokration Hospital, Medical School of Aristotle, University of Thessaloniki, Greece

## Abstract

**Introduction:**

A vermiform appendix in an inguinal hernia, inflamed or not, is known as Amyand's hernia. Here we present a case series of four men with Amyand's hernia.

**Case presentations:**

We retrospectively studied 963 Caucasian patients with inguinal hernia who were admitted to our surgical department over a 12-year period. Four patients presented with Amyand's hernia (0.4%). A 32-year-old Caucasian man had an inflamed vermiform appendix in his hernial sac (acute appendicitis), presenting as an incarcerated right groin hernia, and underwent simultaneous appendectomy and Bassini suture hernia repair. Two patients, Caucasian men aged 36 and 43 years old, had normal appendices in their sacs, which clinically appeared as non-incarcerated right groin hernias. Both underwent a plug-mesh hernia repair without appendectomy. The fourth patient, a 25-year-old Caucasian man with a large but not inflamed appendix in his sac, had a plug-mesh hernia repair with appendectomy.

**Conclusion:**

A hernia surgeon may encounter unexpected intraoperative findings, such as Amyand's hernia. It is important to be prepared and apply the appropriate treatment.

## Introduction

A vermiform appendix in an inguinal hernia sac, with or without appendicitis, is called Amyand's hernia. Claudius Amyand (1660-1740), a French surgeon working at St George's and Westminster hospitals in London, performed the first successful appendectomy in 1735, on an 11-year-old boy who presented with an inflamed, perforated appendix in his inguinal hernia sac. According to the surgeon's descriptions, the patient also had "a fistula between the scrotum and thigh" and the operation proved to be "very complicated and perplexing," as the pathology consisted of a chronically inflamed appendix contained within the inguinal hernia sac, perforated by a previously swallowed pin. At surgery the appendix was removed. The patient eventually recovered and was "discharged with a truss, which he was ordered to wear for some time." The case was published in the Philosophical Transactions of the Royal Society of London [1].

Inguinal hernia repair is one of the most common operations in surgical practice. Despite that, hernias often pose technical dilemmas, even for the experienced surgeon [2]. The surgeon may encounter unusual findings, such as a vermiform appendix partly or fully contained in the hernia sac, inflamed or non-inflamed, stretched or curved, and adhered or not adhered to the sac walls. Whether or not an appendectomy should be performed at the same times as the hernia repair is debatable. The aim of this study is to present the experience of our university surgical department with Amyand's hernias along with a review of the literature on this subject.

## Case presentations

We undertook a retrospective review of the case histories of 963 Caucasian patients with inguinal hernia, admitted and treated in our surgical department over a 12-year period (between 1998 and 2009). Both elective and emergency cases were included in the study. Information was obtained from their medical records and their detailed operative protocols. Four Caucasian patients presented with Amyand's hernia (0.4%). All patients had an uneventful postoperative course, without any recorded postoperative wound infection or hernia recurrence.

### Case 1

A 32-year-old man, with the clinical appearance of an incarcerated right groin hernia, had acute appendicitis and underwent simultaneous appendectomy and conventional modified Bassini hernia repair.

### Case 2

A 36-year-old man, with the clinical appearance of a non-incarcerated right groin hernia, with a normal appendix within his hernia sac, had a mesh-plug hernia repair without appendectomy.

### Case 3

A 43-year-old man, with the clinical appearance of a non-incarcerated right groin hernia, with a normal appendix within his hernia sac, had a mesh-plug hernia repair without appendectomy.

### Case 4

The fourth patient, a 25-year-old man, presented with a 20 cm long but non-inflamed appendix. This was fully contained and adhering to his inguinal sac wall, pulling the cecum up to the internal inguinal orifice. The patient underwent a mesh-plug hernia repair along with appendectomy.

## Discussion

Acute appendicitis within an inguinal hernia accounts for 0.1% of all cases [2-7]. Inflammation of the appendix is attributed to external compression of the appendix at the neck of the hernia. The inflammatory status of the vermiform appendix determines the surgical approach and the type of hernia repair. All surgeons agree that if appendicitis exists, the repair of the hernia should be performed with Bassini or Shouldice techniques, without making use of synthetic meshes or plugs within the defect [2,5,8] due to the high risk of suppuration of such materials.

In the case of a normal appendix, incidentally found within the hernia sac, the performance of a prophylactic appendectomy along with the hernia repair is not favored by many authors [9,10]. Appendectomy adds the risk of infection to an otherwise clean procedure. Superficial wound infection increases morbidity; and deep infection may contribute to hernia recurrence. In addition, surgical manipulation to achieve visualization of the entire appendix and its base, by enlarging the hernial defect or distending the neck of the hernial sac, increases the possibility of recurrence by weakening the anatomic structures around the defect [2,5,7,10]. There are authors who recommend reduction of the appendix and mesh hernioplasty if there is no acute appendicitis, and appendectomy followed by endogenous hernia repair if an inflamed appendix is found [7,10,11]. Although these general rules are certainly acceptable, there are more clinical scenarios to keep in mind. Losanoff and Basson have distinguished four basic types of Amyand's hernias, which should be treated differently (see Table 1 for classification) [4,5].

**Table 1:** Classification of Amyand's hernias after Losanoff and Basson [4,5]

Classification	Description	Surgical Management
Type 1	Normal appendix within an inguinal hernia	Hernia reduction, mesh repair, appendectomy in young patients
Type 2	Acute appendicitis within an inguinal hernia, no abdominal sepsis	Appendectomy through hernia, primary endogenous repair of hernia, no mesh
Type 3	Acute appendicitis within an inguinal hernia, abdominal wall or peritoneal sepsis	Laparotomy, appendectomy, primary repair of hernia, no mesh
Type 4	Acute appendicitis within an inguinal hernia, related or unrelated abdominal pathology	Manage as types 1 to 3 hernia, investigate or treat second pathology as appropriate

The absence of inflammation in Type 1 advocates elective hernioplasty. Using a prosthetic material in such cases carries the expectation of improved longevity of the repair. It avoids tension on the suture lines and circumvents the metabolic problems related to collagen deficiency, which is known to exist in hernia patients. Whether to remove or leave behind a normal appendix in this clinical scenario cannot be determined because no evidence-based information exists. The decision is rather based on common sense, relating to the patient's age, life expectancy, life-long risk of developing acute appendicitis and the size and overall anatomy of the appendix. Pediatric or adolescent patients have a significantly higher risk of developing acute appendicitis and should therefore have their appendices removed, compared to middle-aged or elderly individuals in whom the appendix should probably be left intact [4,5]. Long, curved appendices have a higher risk of inflammation. Additionally a long appendix which stretches the cecum may cause chronic pain if left behind. Manipulations to detach and reduce the appendix in the abdomen may stimulate the inflammatory process. Furthermore, consideration of appendectomy in young patients must take into account the size of the hernia, since prosthetic material is contraindicated but large hernias are more likely to recur if repaired by making use of endogenous tissue only.

The decision is easier in Type 2 hernias, where appendicitis is found, as they should be treated with appendectomy; however the hernia repair should be performed without making use of prosthetic materials. On the other hand, in septic patients with Amyand's hernia Type 3 (acute appendicitis with peritonitis), or Type 4 (acute appendicitis with other pathology), even the hernioplasty may be contraindicated if the patient's condition is poor or life expectancy is limited.

Looking at our case series, in case 1 we decided not to place a mesh, due to the presence of acute inflammation--appendicitis. This guarded the hernia repair from possible future extension of inflammation in the mesh. In contrast, a mesh was placed in cases 2 and 3 with a normal appendix in their sac. However, in these cases, we decided not to proceed with appendectomy, because this additional procedure could lead to potential damage of the plastic hernia repair. In case 4, given the young age of our patient and the long appendix in his sac, we decided that the increased likelihood for appendicitis in the future necessitated an individual appendectomy. Consequently, our recommendation is that the decision to perform an appendectomy or/and use the mesh-plug technique should always be individualized to the patient.

## Conclusion

In conclusion, a hernia surgeon may encounter unexpected intraoperative findings, such as an Amyand's hernia. The decision as to whether one should perform a simultaneous appendectomy and hernia repair is multifactorial. It is important to be aware of all clinical settings and an appropriate and individualized approach should be applied.

## Consent

Written informed consent was obtained from all patients for publication of this case series and any accompanying images. A copy of the written consent is available for review by the Editor-in-Chief of this journal.

## Competing interests

The authors declare that they have no competing interests.

## Authors' contributions

KP, KB, TP performed the procedures. ML obtained the patients' written informed consent to publish the report, conducted the follow-up examinations, analyzed and interpreted the patient data, and wrote part of the manuscript. KP, NS, MB, EP and AT edited and wrote part of the manuscript. KB and TP were major contributors to reviewing and editing the manuscript. AS made the strategic plan and gave the final approval. All authors read and approved the final manuscript.

## References

[B1] Amyand C Of an inguinal rupture, with a pin in the appendix caeci, incrusted with stone; and some observations on wounds in the guts Philos Trans R Soc London 1736 39 329 336

[B2] Ballas K Kontoulis T Skouras C Triantafyllou A Symeonidis N Pavlidis T Marakis G Sakadamis A Unusual findings in inguinal hernia surgery: Report of 6 rare cases Hippokratia 2009 13 3 169 171 19918306 PMC2765295

[B3] De Garengeot RJC Traite des operations de chirugie 1731 2 Paris: Huart 369 371

[B4] Losanoff JE Basson MD Amyand hernia: what lies beneath--a proposed classification scheme to determine management Am Surg 2007 73 12 1288 1290 18186392

[B5] Losanoff JE Basson MD Amyand hernia: a classification to improve management Hernia 2008 12 3 325 326 10.1007/s10029-008-0331-y 18214637

[B6] Llullaku SS Hyseni HS Kelmendi BZ Jashari HJ Hasani AS A pin in appendix within Amyand's hernia in a six-years old boy: case report and review of literature World J Emerg Surg 2010 5 14 10.1186/1749-7922-5-14 20482877 PMC2882903

[B7] Milanchi S Allins AD Amyand's hernia: history, imaging, and management Hernia 2008 12 3 321 322 10.1007/s10029-007-0304-6 17990042

[B8] Livaditi E Mavridis G Christopoulos-Geroulanos G Amyand's hernia in premature neonates: report of two cases Hernia 2007 11 6 547 549 10.1007/s10029-007-0242-3 17541492

[B9] Sharma H Gupta A Shekhawat NS Memon B Memon MA Amyand's hernia: a report of 18 consecutive patients over a 15-year period Hernia 2007 11 1 31 35 10.1007/s10029-006-0153-8 17001453

[B10] D'Alia C Lo Schiavo MG Tonante A Taranto F Gagliano E Bonanno L Di Giuseppe G Pagano D Sturniolo G Amyand's hernia: case report and review of the literature Hernia 2003 7 2 89 91 10.1007/s10029-002-0098-5 12820031

[B11] Salemis NS Nisotakis K Nazos K Savrinou P Tsohataridis E Perforated appendix and periappendicular abscess within an inguinal hernia Hernia 2006 10 6 528 530 10.1007/s10029-006-0132-0 16932844

